# Factors Associated with Delayed or Missed Second-Dose mRNA COVID-19 Vaccination among Persons >12 Years of Age, United States

**DOI:** 10.3201/eid2808.220557

**Published:** 2022-08

**Authors:** Lu Meng, Neil Chandra Murthy, Bhavini Patel Murthy, Elizabeth Zell, Ryan Saelee, Megan Irving, Hannah E. Fast, Patricia Castro Roman, Adam Schiller, Lauren Shaw, Carla L. Black, Lynn Gibbs-Scharf, LaTreace Harris, Terence Chorba

**Affiliations:** Centers for Disease Control and Prevention Atlanta, Georgia, USA (L. Meng, N.C. Murthy, B.P. Murthy, E. Zell, R. Saelee, M. Irving, H.E. Fast, P.Castro Roman, A. Schiller, L. Shaw, C.L. Black, L. Gibbs-Scharf, L. Harris, T. Chorba);; General Dynamics Information Technology Inc., Falls Church, Virginia, USA (L. Meng, R. Saelee);; Stat-Epi Associates, Inc., Ponte Vedra Beach, Florida, USA (E. Zell); Deloitte Consulting LLP, New York, New York, USA (M. Irving);; Booz Allen Hamilton, Inc., McLean, Virginia, USA (A. Schiller)

**Keywords:** COVID-19, 2019 novel coronavirus disease, coronavirus disease, severe acute respiratory syndrome coronavirus 2, SARS-CoV-2, viruses, respiratory infections, zoonoses, 2019-nCoV vaccine mRNA, vaccine delay, mRNA vaccine, vaccine timing, vaccination, Moderna COVID-19 vaccine, vaccines

## Abstract

To identify demographic factors associated with delaying or not receiving a second dose of the 2-dose primary mRNA COVID-19 vaccine series, we matched 323 million single Pfizer-BioNTech (https://www.pfizer.com) and Moderna (https://www.modernatx.com) COVID-19 vaccine administration records from 2021 and determined whether second doses were delayed or missed. We used 2 sets of logistic regression models to examine associated factors. Overall, 87.3% of recipients received a timely second dose (≤42 days between first and second dose), 3.4% received a delayed second dose (>42 days between first and second dose), and 9.4% missed the second dose. Persons more likely to have delayed or missed the second dose belonged to several racial/ethnic minority groups, were 18–39 years of age, lived in more socially vulnerable areas, and lived in regions other than the northeastern United States. Logistic regression models identified specific subgroups for providing outreach and encouragement to receive subsequent doses on time.

In December 2020, the US Food and Drug Administration (FDA) issued Emergency Use Authorizations (EUAs) for the Pfizer BioNTech (https://www.pfizer.com) and Moderna (https://www.modernatx.com) 2-dose primary mRNA COVID-19 vaccine series (*1*,*2*). The Centers for Disease Control and Prevention (CDC) Advisory Committee on Immunization Practices, part of the National Center for Immunization and Respiratory Diseases, prioritized certain populations to be offered the COVID-19 vaccination first, including healthcare personnel, long-term care facility residents, persons >65 years of age, persons 16–64 years of age with high-risk medical conditions, and essential workers (*3*). Starting in March 2021, Pfizer-BioNTech and Moderna COVID-19 vaccines have been available at pharmacies and from other medical practice providers for anyone >16 years of age. In the 1-year period of this analysis, the recommended intervals between the 2 primary doses were 21 days for the Pfizer-BioNTech vaccine and 28 days for the Moderna vaccine (*4*). On May 10, 2021, FDA expanded the EUA for the Pfizer COVID-19 vaccine to include persons 12–15 years of age (*5*). During August–November 2021, FDA approved a series of EUAs: 1 for an additional primary dose for immunocompromised persons and 1 for a booster dose for persons >18 years of age (*6*).

In the summer of 2021, one of every 10 US persons received the first dose of an mRNA COVID-19 vaccine, ≈15 million still had not received the second dose, and many more had received the second dose outside the recommended intervals between doses (*7*). Persons who start the primary series are presumably amenable to initial vaccination but may then either delay completing or may fail to complete the series. Delayed or missed recommended COVID-19 vaccine doses can hamper national efforts to reduce COVID-19–associated illness, hospitalization, and death (*8*–*10*). More information about this population is valuable for addressing second-dose vaccination barriers and devising interventions to increase primary series completion.

To support nationwide COVID-19 immunization efforts, we performed an analysis to identify demographic factors associated with receiving 1 dose of the 2-dose primary mRNA vaccine series but delaying the second dose or not completing the series. The study was reviewed by CDC and conducted consistent with applicable federal law and CDC policy.

## Methods

We analyzed COVID-19 mRNA vaccine administration data among persons >12 years of age in the United States during December 14, 2020–December 31, 2021. US COVID-19 vaccine administration data are reported from jurisdictions, pharmacies, and federal entities to CDC via immunization information systems, the CDC Vaccine Administration Management System, or direct data submission (*11*). De-identified vaccination records from Idaho were reported for persons >18 years of age; all other states, excluding Texas, and the District of Columbia reported vaccination records for persons >12 years of age. For this analysis, so that all persons who had received a first dose had sufficient time to receive a second dose within a conventionally permissible time frame, we included in our analysis all persons >12 years of age who received a first dose of an mRNA vaccine on or before September 30, 2021, which would allow >3 months (October 1, 2021–December 31, 2021) after a first dose to have received a second dose. We matched de-identified first- and second-dose records according to a unique recipient number (not associated with recipients’ personally identified information) assigned by the reporting entity and an 8-12–digit reporting source code. For each recipient, we calculated the number of days between the first and second doses. To enable scheduling considerations and other unintended or systematic delays, we defined receipt of a timely second dose as a second dose administered <42 days after the first dose. We defined a delayed second dose as a second dose administered >42 days after the first dose. Although data on the efficacy of second mRNA COVID-19 vaccine doses administered beyond this window are limited, we chose a cutoff of 42 days because that has been the limit of days between doses conventionally considered permissible when a delay is unavoidable (*4*). We defined a missed second dose as receipt of the first dose but not having a matching second dose on record. We excluded from analysis persons whose records indicated that the second dose was administered earlier than the vaccine brand–specific recommended dosing interval, with a 4-day grace period, which for this study we defined as 17 days (Pfizer-BioNTech) and 24 days (Moderna). We included recipients of both mRNA vaccine brands (e.g., first dose Pfizer-BioNTech and second dose Moderna vaccine). We determined second-dose timing according to brand of the first-dose vaccine.

**Figure 1:**
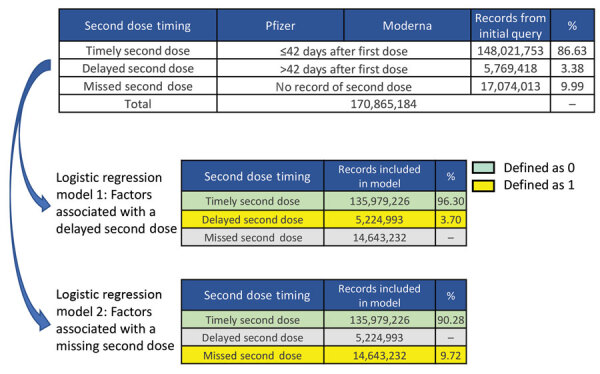
Logistic regression models built to examine sociodemographic factors associated with missed or delayed second doses in primary series of mRNA COVID vaccination among persons >12 years of age, United States. The table at the top includes all records from initial query that met the inclusion criteria. The lower 2 sub-tables provide the number of records included in each of the 2 multivariable logistic regression models. Pfizer-BioNTech, https://www.pfizer.com; Moderna, https://www.modernatx.com.

On the basis of the inclusion criteria and definitions, we attempted to match 323 million mRNA COVID-19 vaccine administration records reported to CDC as doses having been administered during December 14, 2020–December 31, 2021, including 170,865,184 first-dose records issued by September 30, 2021, and 153,791,171 second-dose records issued by December 31, 2021. We excluded 15,017,733 (8.8%) records for which county of residence was missing. We built logistic regression models to examine sociodemographic factors associated with a delayed second dose (model 1) or a missed second dose (model 2) (Figure 1).

### Dependent Variables

In model 1, recipients whose second dose was delayed were classified as 1 and recipients who received a timely second dose were classified as 0. Recipients who did not receive a second dose were excluded from this model.

In model 2, recipients who did not receive a second dose were classified as 1 and recipients who received a timely second dose were classified as 0. Recipients who received a delayed second dose were excluded from this model.

### Independent Variables

**Figure 2:**
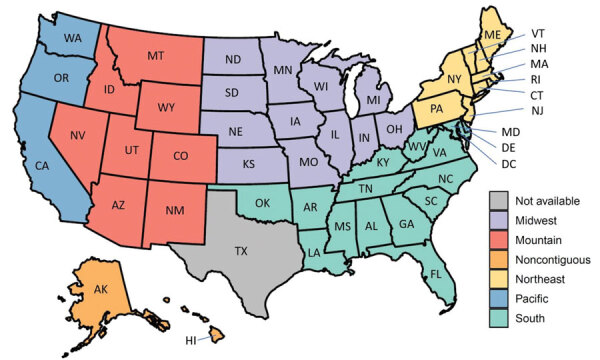
States in each region used in study of factors associated with delayed or missed second-dose mRNA COVID-19 vaccination among persons >12 years of age, United States.

We included in the model the fundamental demographic information reported to CDC in the vaccine administration records, including recipient’s age, sex, race/ethnicity, and postal code. In addition, we derived the CDC/Agency for Toxic Substances and Disease Registry Social Vulnerability Index (SVI) scores and the CDC Urban-Rural Classification scores from vaccination records according to the recipient’s county of residence (*12*,*13*). We generated urban-rural classification and SVI score tertiles of county of residence (low, medium, high) for each record. Higher SVI scores indicated counties that were more socially vulnerable. Independent variables for the 2 logistic regression models included first-dose vaccine type (Pfizer-BioNTech, Moderna), age group (12–17, 18–39, 40–64, >65 years), sex (male, female), race/ethnicity (Hispanic, non-Hispanic Asian/Other Pacific Islander, non-Hispanic Black, non-Hispanic White, non-Hispanic American Indian/Alaska Native, other/unknown), US region of residence (South, Midwest, Mountain, Pacific, Northeast, Noncontiguous [Figure 2]), SVI tertile of county of residence (low, medium, high), and urbanicity (metro, nonmetro). In our results, racial/ethnic groups are reported as Hispanic, Asian, Black, White, American Indian/Alaska Native, and other/unknown.

We based odds ratio (OR) and 95% CI calculations on regression estimates and performed descriptive analyses for all input variables. We included in this study factors for which OR was >1.150 or <0.850. All analyses were conducted in the cloud-based data platform Microsoft Azure DataBricks (https://azure.microsoft.com/en-us/services/databricks).

## Results

In total, we attempted to match 155,847,451 records of first-dose mRNA vaccine receipt containing recipient’s county of residence (91.2% of the total 170,865,184 first-dose records) to records of second-dose mRNA vaccine receipt. We merged those records with SVI and urbanicity variables and included them in the descriptive analyses and multivariable logistic regression models. Of the 155,847,451 first-dose mRNA vaccination records, matching indicated that 135,979,226 (87.3%) persons received a timely second dose and that 5,224,993 (3.4%) received a delayed second dose; 14,643,232 (9.4%) first-dose records lacked a matched second-dose record and the first-dose recipients were considered to have missed the second dose (Table 1). Of all first-dose recipients, 41.9% were White, 11.3% were Hispanic, 7.2% were Black, 4.4% were Asian, 0.7% were American Indian/Alaska Native, and 34.5% were of other/unknown race/ethnicity. Of all recipients, 46.2% were male, and mean (±SD) age was 48.0  (±20.3) years.

**Table 1:** Timing of second dose of a 2-dose primary mRNA COVID-19 vaccine series, by sociodemographic factors, United States, December 14, 2020–December 31, 2021*

Variable	Second dose timing, no. (%)			Total, no. (%)
	Timely†	Delayed‡	Missed§	
Total	135,979,226 (87.25)	5,224,993 (3.35)	14,643,23 (9.40)	155,847,451
Vaccine type, dose 1				
Moderna	53,478,853 (87.04)	2,384,368 (3.88)	5,577,569 (9.08)	61,440,790
Pfizer-BioNTech	82,500,373 (87.39)	2,840,625 (3.01)	9,065,663 (9.60)	94,406,661
Age group, y				
12–17	9,762,549 (86.60)	280,348 (2.49)	1,230,395 (10.91)	11,273,292
18–39	39,414,160 (85.13)	1,621,400 (3.50)	5,264,452 (11.37)	46,300,012
40–64	52,589,787 (88.27)	1,934,658 (3.25)	5,053,236 (8.48)	59,577,681
>65	34,212,730 (88.41)	1,388,587 (3.59)	3,095,149 (8.00)	38,696,466
Sex				
M	62,548,887 (86.85)	2,393,426 (3.32)	7,076,540 (9.83)	72,018,853
F	73,430,339 (87.60)	2,831,567 (3.38)	7,566,692 (9.03)	83,828,598
Urbanicity				
Metro	120,443,535 (87.14)	4,633,895 (3.35)	13,145,421 (9.51)	138,222,851
Nonmetro	15,535,691 (88.15)	591,098 (3.35)	1,497,811 (8.50)	17,624,600
Social Vulnerability Index (*12*)				
High	41,938,563 (85.92)	1,873,526 (3.84)	5,003,810 (10.25)	48,815,899
Medium	54,681,094 (86.36)	2,090,878 (3.30)	6,544,599 (10.34)	63,316,571
Low	39,359,569 (90.04)	1,260,589 (2.88)	3,094,823 (7.08)	43,714,981
Race/ethnicity				
Hispanic	14,678,149 (83.38)	622,364 (3.54)	2,302,543 (13.08)	17,603,056
Black	9,711,675 (86.24)	446,893 (3.97)	1,102,700 (9.79)	11,261,268
American Indian/Alaska Native	860,111 (75.91)	54,520 (4.81)	218,355 (19.27)	1,132,986
Asian/OPI	6,047,962 (88.09)	210,615 (3.07)	607,609 (8.85)	6,866,186
White	58,829,793 (90.17)	1,972,730 (3.02)	4,445,116 (6.81)	65,247,639
Other/unknown	45,851,536 (85.33)	1,917,871 (3.57)	5,966,909 (11.10)	53,736,316
Region				
South	41,188,846 (86.15)	1,775,680 (3.71)	4,844,461 (10.13)	47,808,987
Midwest	28,738,970 (91.57)	811,360 (2.59)	1,832,846 (5.84)	31,383,176
Mountain	11,446,355 (86.39)	574,949 (4.34)	1,227,706 (9.27)	13,249,010
Pacific	27,077,135 (83.19)	1,254,754 (3.86)	4,216,649 (12.95)	32,548,538
Noncontiguous	517,084 (74.17)	28,439 (4.08)	151,668 (21.75)	697,191
Northeast	27,010,836 (89.56)	779,811 (2.59)	2,369,902 (7.86)	30,160,549

*Pfizer-BioNTech, https://www.pfizer.com; Moderna, https://www.modernatx.com. OPI, other Pacific Islander. †Second dose administered <42 d after first dose. ‡Second dose administered >42 d after second dose. §Received the first dose but no matching second dose on record.

Model 1 of the 2 logistic regression models (Table 2) shows the results of the logistic regression model examining factors associated with a delayed second dose, conditional on receiving a second dose. Model 1 analyzed a total of 141,204,219 matched first- and second-dose pairs; of those, 135,979,226 (96.3%) first-dose recipients received a timely second dose and 5,224,993 (3.7%) received a delayed second dose. Compared with initial Pfizer-BioNTech vaccine recipients, initial Moderna vaccine recipients were more likely to have received a delayed second dose (OR 1.267, 95% CI 1.265–1.270). Recipients 18–39 years of age were more likely to have received a delayed second dose compared with recipients 12–17 years of age (OR 0.763, 95% CI 0.759–0.766). Compared with recipients who resided in low SVI tertile counties, those in high SVI tertile counties were more likely to have received a delayed second dose (OR 1.198, 95% CI 1.196–1.201). Compared with White recipients, delayed receipt of a second dose was more likely among American Indian/Alaska Native (OR 1.508, 95% CI 1.494–1.522), Black (OR 1.310, 95% CI 1.305–1.314), and Hispanic (OR 1.172, 95% CI 1.168–1.175) persons. Compared with recipients who resided in counties in the Northeast, delayed receipt of a second dose was more likely among persons who resided in counties in the South (OR 1.425, 95% CI 1.421–1.429), Pacific (OR 1.507, 95% CI 1.503,1.512), Noncontiguous (OR 1.886, 95% CI 1.863–1.909), and Mountain (OR 1.648, 95% CI 1.641–1.655) regions.

**Table 2:** Logistic regression models examining sociodemographic factors associated with delayed or missed second dose of a 2-dose primary mRNA COVID-19 vaccine series, United States, December 14, 2020–December 31, 2021*

Variable	Model 1			Model 2	
	Delayed second dose,† n = 141,204,219			Missed second dose,‡ n = 155,847,451	
	Coefficient estimate	OR (95% CI)		Coefficient estimate	OR (95% CI)
Vaccine type, dose 1					
Moderna	0.237	1.267 (1.265–1.270)§		−0.012	0.988 (0.987–0.989)
Pfizer-BioNTech	Referent			Referent	
Age group,y					
12–17	−0.271	0.763 (0.759–(0.766)§		−0.064	0.938 (0.936–0.940)
40–64	−0.112	0.894 (0.892–0.896)		−0.301	0.740 (0.739–0.741)§
>65	−0.008	0.992 (0.990–0.995)		−0.298	0.743 (0.741–0.744)§
18–39	Referent			Referent	
Sex					
F	0.006	1.006 (1.004–1.008)		−0.082	0.922 (0.921–0.923)
M	Referent			Referent	
Urbanicity					
Metro	0.048	1.049 (1.046–1.052)		−0.038	0.963 (0.961–0.965)
Nonmetro	Referent			Referent	
Social Vulnerability Index (*12*)					
High	0.181	1.198 (1.196–1.201)§		0.155	1.168 (1.166–1.170)§
Medium	0.092	1.096 (1.094–1.099)		0.260	1.297 (1.295–1.299)§
Low	Referent			Referent	
Race/ethnicity					
Hispanic	0.159	1.172 (1.168–1.175)§		0.560	1.751 (1.748–1.754)§
Black	0.270	1.310 (1.305–1.314)§		0.320	1.377 (1.373–1.380)§
American Indian/Alaska Native	0.411	1.508 (1.494–1.522)§		1.015	2.760 (2.746–2.774)§
Asian/OPI	0.010	1.010 (1.005–1.015		0.128	1.137 (1.134–1.140)
Other/unknown	0.117	1.124 (1.122–1.127)		0.397	1.487 (1.485–1.489)§
White	Referent			Referent	
Region					
South	0.354	1.425 (1.421–1.429)§		0.277	1.319 (1.317–1.322)§
Midwest	−0.007	0.993 (0.990–0.996)		−0.246	0.782 (0.780–0.783) §
Mountain	0.499	1.648 (1.641–1.655)§		0.070	1.072 (1.069–1.075)
Pacific	0.410	1.507 (1.503–1.512)§		0.448	1.566 (1.563–1.568)§
Noncontiguous	0.634	1.886 (1.863–1.909)§		1.111	3.038 (3.020–3.056)§
Northeast	Referent			Referent	

*Pfizer-BioNTech, https://www.pfizer.com; Moderna, https://www.modernatx.com. OPI, Other Pacific Islander; OR, odds ratio. †Second dose administered >42 d after first dose. This model examins factors associated with a delayed second dose, conditional on receiving a second dose. ‡Received the first dose but no matching second dose on record. §Factors with OR >1.150 or <0.850.

Model 2 of the 2 logistic regression models (Table 2) shows the result of the model examining factors associated with a missed second dose. Model 2 analyzed a total of 150,622,458 first-dose records; of these, 135,979,226 (90.3%) first-doses recipients receieved a timely second dose and 14,643,232 (9.7%) missed the second dose. First-dose recipients 18–39 years of age were more likely to have missed the second dose compared with those 40–64 (OR 0.740, 95% CI 0.739–0.741) and >65 (OR 0.743, 95% CI 0.742–0.744) years of age. Compared with first-dose recipients who resided in counties in the low SVI tertile, the second dose was more likely to have been missed by persons in medium (OR 1.297, 95% CI 1.295–1.299) and high SVI tertile counties (OR 1.168, 95% CI 1.166–1.170). Compared with White first-dose recipients, the second dose was more likely to have been missed by American Indian/Alaska Native (OR 2.760, 95% CI 2.746–2.774), Black (OR 1.377, 95% CI 1.373–1.380), Hispanic (OR 1.751, 95% CI 1.748–1.754) recipients, and those of other or unknown race (OR 1.487, 95% CI 1.485–1.489). Compared with first-dose recipients who resided in counties in the Northeast, the second dose was more likely to have been missed by persons who resided in counties in the Pacific (OR 1.566, 95% CI 1.563–1.568), Noncontiguous (OR 3.038, 95% CI 3.020–3.056), and South (OR 1.319, 95% CI 1.317–1.322) regions.

## Discussion

By building models based on millions of US vaccination records to analyze sociodemographic factors associated with delayed or missed second doses in a 2-dose primary mRNA COVID-19 vaccine series, we identified population subgroups that might benefit from targeted interventions aimed at encouraging timely receipt of a second dose and at increasing access and demand for a booster dose. Findings from this study (Table 1) align with previous US reports that nearly 1 in 10 persons who began a 2-dose COVID-19 mRNA vaccination series had not received a second dose (*7*).

Compared with first-dose recipients 18–39 years of age, recipients 40–64 and >65 years of age were less likely to have missed a second dose. Persons in older age groups had more time to complete their primary series, given the prioritization when COVID-19 vaccine first became available. Older adults also are at higher risk for severe COVID-19 illness and may have been more motivated to become fully vaccinated (*14*,*15*). Compared with persons 18–39 years of age, persons 12–17 years of age were less likely to have received a delayed second dose. On May 10, 2021, EUA was granted for COVID-19 vaccine use in persons 12–15 years of age, and peak adolescent vaccination rates were observed during summer 2021, immediately before the start of the 2021–22 school year (*16*,*17*). Lower rates of delayed second-dose vaccine receipt by those 12–17 years of age may have partially resulted from parent and child desire to return to in-person learning and from vaccination encouragement by or mandates from schools (*18*–*20*).

In our analyses, receipt of the second vaccine dose was more likely to have been delayed by initial Moderna vaccine recipients than by initial Pfizer-BioNTech recipients. In the context of our analysis, given the shorter recommendation period for the second dose of Pfizer-BioNTech vaccine, the definition of delayed second dose (>42 days) gave Pfizer recipients a window of 7 more days (beyond the vaccine-specific recommended dosing interval) than Moderna recipients to complete a second dose, which might contribute to the result. Previous studies suggested that Moderna recipients were more likely to have experienced side effects (especially after the second dose) than were Pfizer-BioNTech recipients (*21*,*22*). Fear of side effects may also have contributed to the higher percentage of delayed second doses among Moderna recipients (*23*).

When compared with first-dose recipients who were White, members of several racial/ethnic minority groups were more likely to have delayed or missed the second dose, including Hispanic, Black, and American Indian/Alaska Native; this finding is consistent with reports from other studies that uptake of COVID-19 vaccination and other vaccinations was lower among members of these minority groups (*24*–*26*). Many factors may contribute to this disparity. Poverty rates are higher among Black (19.5%) and Hispanic (17.0%) than among White (8.2%) persons (*27*). Lower-income persons may be concerned about taking time off work to get vaccinated and to recuperate should they experience side effects (*28*,*29*). In addition, this racial/ethnic disparity may in part reflect vaccine access barriers for getting a timely second dose among Black and Hispanic persons (*30*). In comparison, Asian first-dose recipients were more likely to receive a timely second dose, which may reflect lower vaccine hesitancy observed among this group (*24*). These observations all highlight the value of knowing which barriers prevent timely second-dose completion for racial/ethnic minority recipients.

Delayed or missed second doses were less likely among those who reside in low SVI tertile counties than among those who reside in high SVI tertile counties. SVI comprises 4 themes (socioeconomic status, household composition and disability, minority status and language, and housing and transportation) constructed by using 15 social and environmental variables from the US Census (*13*). Lower SVI scores indicate that an area is less socially vulnerable. Previous research found that COVID-19 vaccine coverage was lower in rural counties than in urban counties (*31*). Residents of communities within more socially vulnerable areas may have more barriers to accessing vaccination providers, including limited transportation options, higher disability, and reduced ability to seek out or engage with vaccine providers (*32*,*33*). Technologic disparities and reduced health literacy resulting from language and education barriers could contribute to the finding that those able to receive a first dose missed or delayed receipt of their second dose, especially if challenges involved accessing information regarding vaccine availability or scheduling a second dose within the appropriate time interval (*34*).

Recipients who resided in counties in the Northeast region were more likely than recipients in other regions to receive a timely second dose and less likely to miss their second dose. The Northeast region has reported the highest vaccination coverage since vaccine availability, which may in part reflect the fact that this region has the highest per capita income and the lowest percentage of uninsured persons, both of which have been correlated with vaccination coverage (*35*). The population in the Northeast region is also older and has more college graduates, which are 2 population characteristics associated with lower vaccine hesitancy and higher COVID-19 vaccination coverage (*36*,*37*). In addition, compared with other US regions, Northeast jurisdictions promoted vaccinations at different administrative levels (e.g., local or state), including vaccine mandates and proof-of-vaccination requirements for indoor dining, indoor entertainment venues, and large gatherings (*38*,*39*). Such policies and cultural norms may also have contributed to populations receiving their second vaccine dose on time.

First among the limitations of our study, a small percentage of records submitted to CDC lacked recipients’ county of residence information (8.8%), which in some states contributed to the loss of sample size for generation of SVI and urbanicity measures. Race/ethnicity data were missing on 30% of records, which may affect the accuracy of findings related to race/ethnicity. Race/ethnicity information is not a customary unit of data gathered when arranging vaccination appointments, unlike age (date of birth) or sex. Missing race/ethnicity data result in part from data-reporting limitations in some counties and states and from incomplete data collection/reporting at the beginning of vaccine rollout. Second, in our analysis, identifying second-dose recipients depended on the link between vaccination records in jurisdiction-specific immunization information systems. Persons who received a second dose in a different jurisdiction from that of their first dose or for whom we were unable to match first and second primary series doses could not be represented accurately in these results. Third, persons who received the first dose in the fall of 2021 (e.g., September) had fewer months in which to receive their second dose and be matched than did those who received their first dose early in 2021 (because of our cutoff of December 31, 2021). Thus, we would not have captured any delayed second doses for those who received their second dose in 2022 (i.e., after the cutoff); this population would have been defined as having missed the second dose and was included in the model examining risk factors associated with having missed the second dose. Fourth, characteristics other than the sociodemographic factors that we analyzed could have been associated with series completion.

Because vaccine effectiveness against infection and hospitalization has been found to be higher with an extended (6–8-week) interval than with a standard (3–4-week) interval (*40*), CDC provided guidance in March 2022 that an 8-week interval might be optimal for some persons, especially for men and boys 12–39 years of age (*41*). Future studies of delayed second-dose completion of the mRNA primary series should consider that newer recommended intervals between first and second doses may be longer than the intervals that we considered here.

Our study highlights demographic factors associated with delayed or missed second doses in the 2-dose primary series of mRNA COVID-19 vaccine in the United States and identifies population subgroups that may benefit from outreach and encouragement to receive subsequent doses on time. Second doses were more likely to be delayed or missed for members of several racial/ethnic minority groups, persons <40 years of age, persons living in more socially vulnerable or nonmetro areas, and persons living in regions other than the Northeast. Interventions and efforts addressing social and health inequalities and promoting vaccine-related policies can potentially increase access and demand for COVID-19 vaccine and improve subsequent dose completion.
